# Impact of Lime Saturation Factor on Alite-Ye’Elimite Cement Synthesis and Hydration

**DOI:** 10.3390/ma17123035

**Published:** 2024-06-20

**Authors:** Xiaodong Li, Bing Ma, Wenqian Ji, Shang Dou, Hao Zhou, Houhu Zhang, Jiaqing Wang, Yueyang Hu, Xiaodong Shen

**Affiliations:** 1 Nanjing Institute of Environmental Sciences, Ministry of Ecology and Environment of the People’s Republic of China, Nanjing 210042, China; lixiaodong@nies.org (X.L.); myyanb@aliyun.com (B.M.); 2 College of Materials Science and Engineering, Nanjing Tech University, Nanjing 211816, China; doushang12@163.com (S.D.); xdshen@njtech.edu.cn (X.S.); 3 College of Chemical Engineering, Nanjing Tech University, Nanjing 211816, China; jwq@njtech.edu.cn; 4 College of Civil Engineering, Nanjing Forestry University, Nanjing 210037, China; jiaqingw@njfu.edu.cn; 5 College of Materials Science and Engineering, Yancheng Institute of Technology, Yancheng 224051, China; huyueyang1989@163.com

**Keywords:** C_4_A_3_$ and C_3_S coexist, lime saturation factor (KH), secondary thermal treatment, hydration

## Abstract

Alite(C_3_S)-Ye’elimite(C_4_A_3_$) cement is a high cementitious material that incorporates a precise proportion of ye’elimite into the ordinary Portland cement. The synthesis and hydration behavior of Alite-Ye’elimite clinker with different lime saturation factors were investigated. The clinkers were synthesized using a secondary thermal treatment process, and their compositions were characterized. The hydrated pastes were analyzed for their hydration products, pore structure, mechanical strength, and microstructure. The clinkers and hydration products were characterized using XRD, TG-DSC, SEM, and MIP analysis. The results showed that the Alite-Ye’elimite cement clinker with a lime saturation factor (KH) of 0.93, prepared through secondary heat treatment, contained 64.88% C_3_S and 2.06% C_4_A_3_$. At this composition, the Alite-Ye’elimite cement clinker demonstrated the highest 28-day strength. The addition of SO_3_ to the clinkers decreased the content of tricalcium aluminate (C_3_A) and the ratio of Alite/Belite (C_3_S/C_2_S), resulting in a preference for belite formation. The pore structure of the hydrated pastes was also investigated, revealing a distribution of pore sizes ranging from 0.01 to 10 μm, with two peaks on each differential distribution curve corresponding to micron and sub-micron pores. The pore volume decreased from 0.22 ± 0.03 to 0.15 ± 0.18 cm^3^ g^−1^, and the main peak of pore distribution shifted towards smaller sizes with increasing hydration time.

## 1. Introduction

Cement is the one of the most irreplaceable and ubiquitous man-made materials in modern society [1]. In 2020, China accounted for 57% of global cement production with a production volume of about 2.37 billion tonnes [2,3]. However, sintering limestone and clay during cement manufacturing releases a huge amount of carbon dioxide (CO_2_), contributing about 6% to global CO_2_ emission [4]. In light of the global emphasis on carbon reduction, the cement industry, which is recognized as a major source of carbon emissions, is facing considerable pressure to attain carbon peak and carbon neutrality objectives.

Recent studies on carbon reduction in cement clinker have mainly focused on the development of low-calcium clinker and the improvement of clinker quality to reduce the clinker factor. The development of low-calcium clinker primarily revolves around the design of belite as the main mineral phase, including the study of belite-sulfoaluminate cement [5,6] and belite-sulfoaluminate-sulfosilicate cement [7,8], among others. In terms of improving clinker quality to reduce the clinker factor, research mainly revolves around the modulation of alite structure and the introduction of early strength minerals. This includes modifying the alite structure from the M3-type to M1-type [9,10] or R-type [11] through ion doping and introducing ye’elimite in the clinker, thus developing Alite-Ye’elimite cement [12,13].

In general, ordinary Portland cement exhibits excellent late strength performance but unsatisfactory early strength, while sulfoaluminate cement [14] is just the opposite. Alite-Ye’elimite cement (Alite Calcium sulfoaluminate cement, AC$A) is a solution to make up for the above two cements’ shortage [15]. AC$A is an innovative cement that incorporates an appropriate amount of ye’elimite (C_4_A_3_$) into cement clinker. Ye’elimite is a mineral that exhibits high early strength and can reduce the shrinkage rate of ordinary Portland cement [16], as well as improve physical properties such as frost resistance, impermeability, and durability [12,17]. More than the common orthorhombic phase, a recent study synthesized cubic-phase ye’elimite by doped iron [18,19]. In AC$A, ye’elimite significantly contributed to the 1 d and 3 d early strength of cement, while alite is essential for achieving a high 28 d strength. Ye’elimite-rich cement has potential to benefit the environment by replacing or being combined with ordinary Portland cement [13].

Alite forms rapidly at temperatures of about 1450 °C while ye’elimite mostly decomposes above 1350 °C. An approach for producing AC$A clinker, involving two successive sintering steps, has been reported [12]. Recently, some researchers have even successfully produced the clinker at temperatures as high as 1300 °C [20].

In clinker production, MgO and SO_3_ were found to be the most common foreign oxides. It has been reported that the presence of MgO enhances the formation of M3 alite, whereas SO_3_ can transform M3 alite into M1 alite [10,21,22,23]. Modifying the MgO/SO_3_ ratio can stabilize M1 alite, and cement containing M1 alite exhibits a 10% increase in mechanical strength compared to those containing M3 alite [21]. SO_3_ has the ability to stabilize M1 alite independently, regardless of the presence of other foreign ions [22]. What is more, the gaseous SO_2_ can enhance the content of M1 alite in secondary sintered cement clinker [12].

The parameters of lime saturation factor (KH) = (CaO − 1.65A1_2_O_3_ − 0.35 Fe_2_O_3_)/(2.8SiO_2_), silica ratio (SR) = SiO_2_/(Al_2_O_3_ + Fe_2_O_3_), and alumina ratio (AR) = Al_2_O_3_/Fe_2_O_3_ are widely used for the design of clinker sintering [24]. In this work, we maintained constant values of the SR and AR parameters while varying the KH levels (low, medium, and high) for clinker synthesis. The mineral composition of the clinker and its hydration properties were thoroughly examined. The objective was to determine the optimal KH level that would yield AC$A cement with superior compressive strength at both early and late ages.

Overall, this study provides valuable insights into the synthesis and hydration of Alite-Ye’elimite cement clinkers and highlights the importance of optimizing the composition for achieving the best mechanical strength properties.

## 2. Materials and Methods

### 2.1. Sample Preparation

The AC$A clinkers were synthesized using the procedures described in previous studies [12,25]. Industrial limestone, fly ash, clay, and silica were milled to achieve a particle size that could pass through an 80 μm sieve. The chemical compositions of these materials are shown in Table 1. Analytical-grade MgO, K_2_O, and CaSO_4_·2H_2_O from Sigma-Aldrich (St. Louis, MO, USA) were used without any additional treatment.

**Figure 1:**
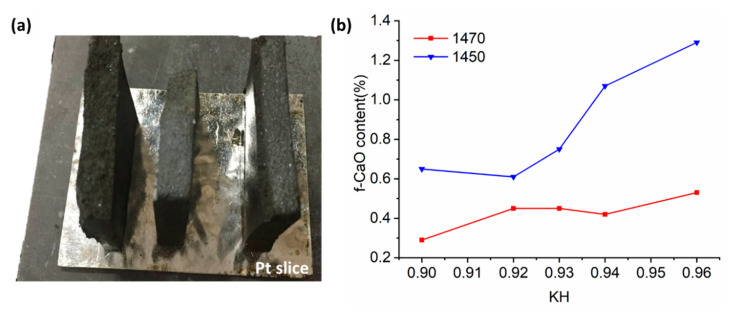
Clinker disks sintered on Pt slice (**a**) and the comparison of f-CaO contents at two sintering temperatures (**b**).

The clinker modulus was designed with specific values for SR (2.50), AR (3.10), and KH (0.90, 0.93, and 0.96), with labels LKH, MKH, and HKH, respectively. The ratios of the raw meals used are presented in Table 2. After being subjected to dry milling for 12 h, the raw mixtures were compressed into disks measuring 60 mm in diameter and 60 mm in height, using a pressure of 30 MPa and 4 wt% of water. These disks were then dried and sintered in a resistance furnace. The temperature of the furnace was calibrated using Ferro PTCR-MTH testing rings (Mayfield Heights, OH, USA). The raw disks were placed on a Pt slice (Figure 1a) and heated at a rate of 10 °C/min. They were calcinated at 900 °C for 30 min and sintered at either 1450 °C or 1470 °C for a duration of 60 min. After sintering, the clinkers were cooled using forced air. In order to achieve the coexistence of alite and ye’elimite, the cooled clinkers were further annealed in a preheated furnace at a temperature of 1270 °C for 1 h, followed by cooling with forced air. The presence of SO_3_-containing clinker led to the bending of the disks due to the formation of liquid during calcination.

The f-CaO contents were measured in accordance with the Chinese standard (GB/T 176-2017) [26] to assess the firing condition of the clinkers. Figure 1b illustrates the f-CaO content with respect to KH at two different sintering temperatures. It is noteworthy that all the clinkers, sintered at 1470 °C, had less than 0.5% f-CaO content, which is the designated sintering temperature for the subsequent study. For further characterization and hydration experiments, the clinker blocks were finely ground using a tungsten carbide vibrating mill for two cycles of 10 s each.

In order to investigate hydration, pastes were prepared using clinker powders, 4 wt.% of gypsum, and DI water with a water-to-cement ratio (*w*/*c*) of 0.35. The ingredients were stirred for 60 s and then transferred into centrifuge tubes for setting at a temperature of 20 ± 1 °C and a relative humidity of 95%. The hydration process was halted at specific time intervals using the solvent exchange method. The samples were immersed in ethanol for a duration of 3 days with three replacements of the solvent. The hardened pastes were then crushed and ground into powders for further analysis.

### 2.2. Characterization

#### 2.2.1. X-ray Diffraction and Rietveld Quantitative Analysis

XRD measurements were conducted using a Rigagu Miniflex workstation with Cu Kα radiation (λ = 0.154059 nm). The X-ray tube operated at 40 kV and 15 mA. Data were collected from 5° to 70° (2θ) over a span of 13 min, with a step size of 0.01°. Rietveld quantitative analysis was performed using GSAS 1.0 [27] EXPGUI [28]. The crystal structures used for refinement included C_3_S (Alite, ICSD 64759), C_2_S (Belite, ICSD 79553), C_4_AF (Brownmillerite, ICSD 2841), C_3_A (tricalcium aluminate ICSD 1841), C_4_A_3_$ (Ye’elimite, ICSD 9560), and f-CaO (Free lime, ICSD 52783). Background and profile fittings were accomplished using shifted Chebyshev polynomials and Pseudo-Voigt functions. The refinement parameters consisted of phase fractions, lattice parameters, and profiles (GU, GV, and GW).

#### 2.2.2. Microscopy

The clinker blocks were embedded in a two-component epoxy and sequentially polished with sandpapers of 600, 1200, 3000, and 5000 #. To etch the clinker surface, a 1% HNO_3_ alcohol solution was used for a duration of 10 s. Microscopic images were captured using a Leica Microsystems GmbH microscope (Wetzlar, Germany) equipped with a Leica DFC 480 camera.

#### 2.2.3. Particle Size

The particle size distribution (PSD) of clinker powders was measured using the Malvern MS2000 laser diffraction granulometry (Malvern, UK). The powders were dispersed in ethanol using ultrasonication for 60 s. The refractive indices of the clinker and ethanol were set to 1.70 and 1.36, respectively.

#### 2.2.4. Compressive Strength Tests

The mortars were prepared following the Chinese National Standard (GB/T 1346-2011) [29]. The weight ratio of AC$A cement/sand/water was set at 1/3/0.5. The mortars were then placed in molds (40 × 40 × 160 mm^3^) and kept at a temperature of 20 ± 1 °C and a relative humidity of 95% for 24 h. Subsequently, the mortars were removed from the molds and submerged in water at 20 °C until they were ready for strength testing. The compressive strengths were tested in accordance with GB/T 17671-2021 [30] using an automatic cement strength testing machine (AEC 201). The machine had a maximum load capacity of 200 kN and applied a constant loading rate of 2.4 kN s^−1^. Each compressive strength value was the average of four tested specimens. Furthermore, industrial ordinary Portland cement (Type PII 52.5 according to Chinese standards) from Jiangnan Xiaoyetian Cement Co. (Nanjing, China) was used as a reference for the strength testing [25].

#### 2.2.5. Calorimetry

Isothermal calorimetry was performed using an eight-channel Thermometric TAM air Instrument (Los Angeles, CA, USA). Pastes with a water-to-cement ratio (*w*/*c*) of 0.5 were prepared in plastic ampoules and externally mixed before the calorimetry test. The hydration heat release of the pastes was recorded for 72 h at a temperature of 20 °C.

#### 2.2.6. Mercury Intrusion Porosimetry (MIP)

Mercury Intrusion Porosimetry (MIP) was performed using a Poremaster GT-60 instrument (Quantachrome, Boynton Beach, FL, USA) to characterize the porosity and pore size distribution of the hydration pastes. Cracked pieces with regular shapes, which were free from contact with the mold, were selected for the measurement.

#### 2.2.7. Scanning Electron Microscope (SEM)

The microstructures of the hydration pastes were observed using a scanning electron microscope (SEM, Model JSM-5900, JEOL Co., Akishima, Japan). The specimens were fractured and attached to the specimen holder using carbon tapes. Subsequently, a thin layer of gold was applied to enhance electrical conductivity.

## 3. Results and Discussion

### 3.1. Clinkers Sintering

**Figure 2:**
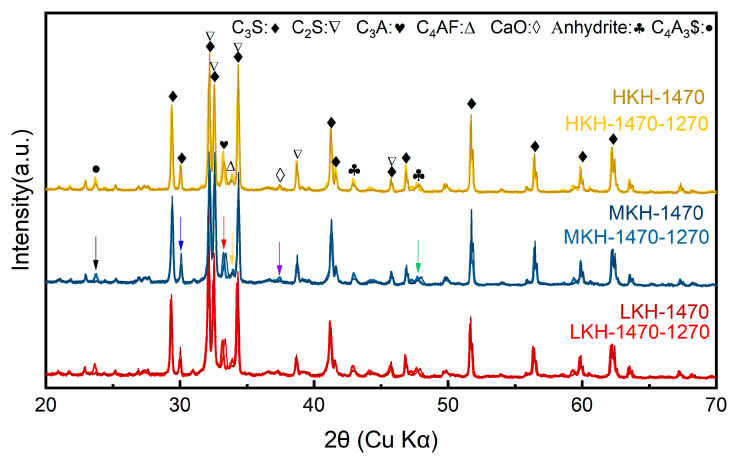
XRD spectra of clinkers.

The mineral compositions of the clinkers were quantitatively characterized using XRD Rietveld analysis, as shown in Figure 2 and Table 3. Three lime saturation factors were compared for clinkers sintered at 1470 °C and subsequently annealed at 1270 °C (darker and brighter profiles in Figure 2). Upon calcination at 1470 °C, four major compositions—C_3_S, C_2_S, C_3_A, and C_4_AF—were obtained from the raw oxides, based on Equations (1)–(4).

(1) $$3Ca{CO}_{3}+SiO_{2}\to {Ca}_{3}SiO_{5}+{3CO}_{2}↑$$

(2) $$2Ca{CO}_{3}+SiO_{2}\to {Ca}_{2}SiO_{4}+{2CO}_{2}↑$$

(3) $$3Ca{CO}_{3}+{Al}_{2}O_{3}\to {Ca}_{3}{Al}_{2}O_{6}+{3CO}_{2}↑$$

(4) $$4Ca{CO}_{3}+{Fe}_{2}O_{3}+{Al}_{2}O_{3}{\to {Ca}_{4}Al}_{2}{Fe}_{2}O_{10}+{4CO}_{2}↑$$

The spectra show notable changes after annealing, indicated by colorful arrows (Black, blue, red, yellow, purple, and green arrows represent the changes of C_4_A_3_$, C_3_S, C_3_A, C_4_AF, and anhydrite, respectively). The thermal treatment results in the formation of C_4_A_3_$ as described in Equation (5) (black arrow in Figure 2) [12]. Additionally, a leftward shift of the fingerprint peaks for C_3_A and C$ is observed due to their reaction (red and green arrows in Figure 2). The decomposition of C_3_S, as shown in Equation (6), is also evident (blue arrow in Figure 2). Although both processes yield CaO, a slight increase is only observed in the spectra of MKH (purple arrow in Figure 2). Based on the chemical compositions of the raw meals (Table 2), the expected content of C_4_AF in the clinkers should be approximately 6% when all Fe_2_O_3_ forms ferrite. However, this value is substantially higher than that observed for the clinkers sintered at 1470 °C. The noticeable increase in C_4_AF content at 1270 °C indicates the reformation of ferrite from the amorphous interphase, as explained in Equation (7) (yellow arrow in Figure 2), which also accounts for the minimal changes in CaO content.

(5) $$3{Ca}_{3}{Al}_{2}O_{6}+{CaSO}_{4}{\to {Ca}_{4}Al}_{6}O_{12}({SO}_{4})+6CaO$$

(6) $${Ca}_{3}SiO_{5}\to {Ca}_{2}SiO_{4}+CaO$$

(7) $${{Ca}_{3}{Al}_{2}O_{6}+Fe}_{2}O_{3}{+CaO\to {Ca}_{4}Al}_{2}{Fe}_{2}O_{10}$$

The impact of SO_3_ dosage on the stabilization of C_2_S at the expense of C_3_S [31] can be mitigated by the addition of MgO [22]. The presence of iron enhances the formation of ye’elimite and clinker, thereby promoting the rapid decomposition of ye’elimite and the volatilization of sulfur [13]. The quantitative changes in mineral content are presented in Table 3, which align with the observed reactions after annealing. The R_wp_ values for all the Rietveld refinements are below 12%. As the lime saturation factor increases, the content of C_3_S increases while that of C_2_S decreases. However, there is little correlation between ye’elimite and the lime saturation factor.

**Figure 3:**
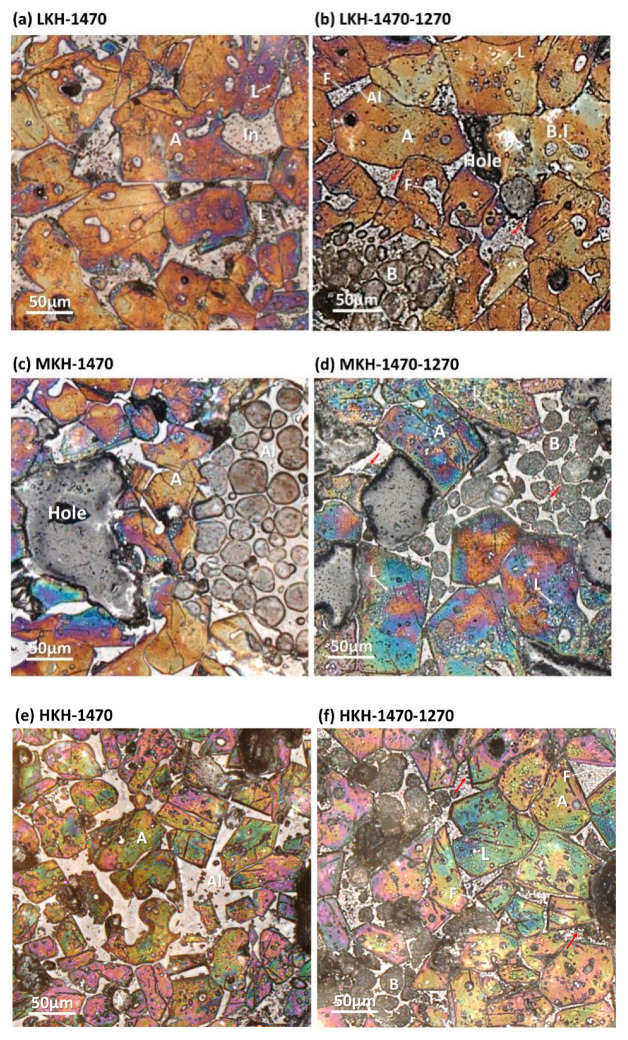
Microscopy images of clinkers before and after secondary annealing at 1270 °C. A is alite, B is belite, Al is aluminate, F is ferrite, L is f-CaO, In is interstitial phases, and B.I is belite inclusion.

Figure 3 illustrates the microscopic images of clinkers, revealing euhedral to subhedral alite, oval belite, and interstitial phases uniformly distributed throughout. These characteristics indicate favorable sintering conditions. With an increase in the lime saturation factor, the alite grain size decreases and becomes fragmented. The belite grains exhibit multidirectional lamellae (B in Figure 3d). Following annealing at 1270 °C, crystal grains with well-defined edges become coarsely crystalline with indistinct boundaries. This observation corroborates Li’s findings [32], which suggest that the decomposition of alite initiates at crystal boundaries. Furthermore, the decomposition of alite granules leads to the formation of belite inclusions (B.I in Figure 3b) and the generation of significant amounts of free lime in the center of the alite (L in Figure 3d). Reflective ferrite appears prominently in the interstitial phases (F in Figure 3b), intermingled with gray aluminate. The dark dots observed in the interstitial phase may indicate the re-formed ye’elimite (red arrows in Figure 3b,d,f) [25]. The microscopy findings demonstrate the decomposition of alite and the formation of ferrite and ye’elimite, which align with the quantitative results obtained from XRD Rietveld analysis.

### 3.2. Hydration Properties of AC$A Cement

**Figure 4:**
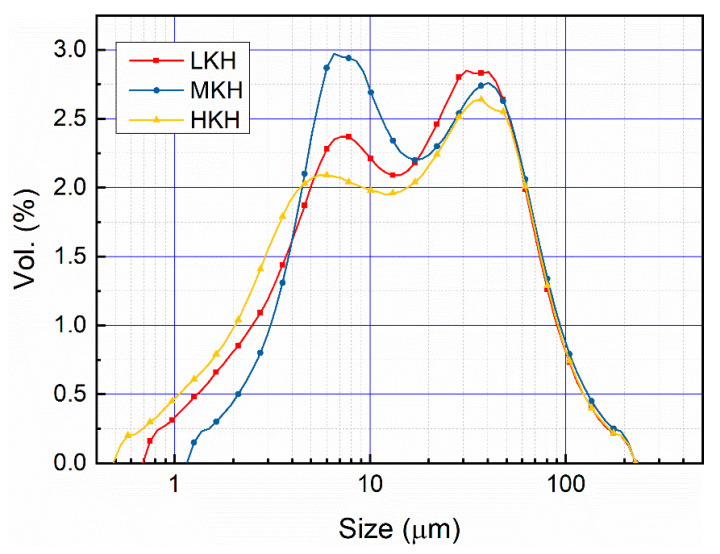
Particle size distribution of clinker powders.

The distribution of particle size has a significant influence on the hydration properties of cement [33]. The hydration investigation involved grinding clinkers with varying lime saturation factors using a standardized protocol. This process resulted in similar particle size distributions, characterized by two peak regions at 5–10 and 20–30 μm (as depicted in Figure 4). The resulting clinker powders of three samples, exhibiting a consistent particle size distribution, were employed to assess the compressive strength of mortars and the hydration characteristics of pastes.

**Figure 5:**
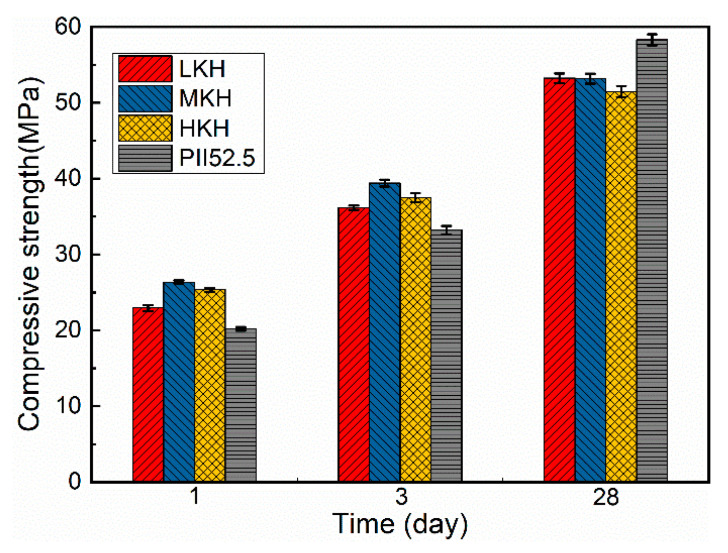
Compressive strength of the mortars.

In Figure 5, it can be observed that MKH exhibits the highest overall compressive strength. The presence of ye’elimite in AC$A cement leads to increased early compressive strength compared to OPC [25]. Typically, PII 52.5 cement achieves compressive strengths of approximately 20 MPa at 1 day and 30 MPa at 3 days. Comparatively, MKH demonstrates a 35.0% increase in compressive strength at 1 day and a 26.7% increase at 3 days when compared to PII 52.5 cement. Although the compressive strengths of AC$A cements at 28 days are lower than PII 52.5 cement, they still exceed 50 MPa. It is important to note that a higher lime saturation, which corresponds to a higher C_3_S content in the clinker (Table 3), does not necessarily result in higher compressive strength.

### 3.3. Microscopy Structure of Hydration Pastes

**Figure 6:**
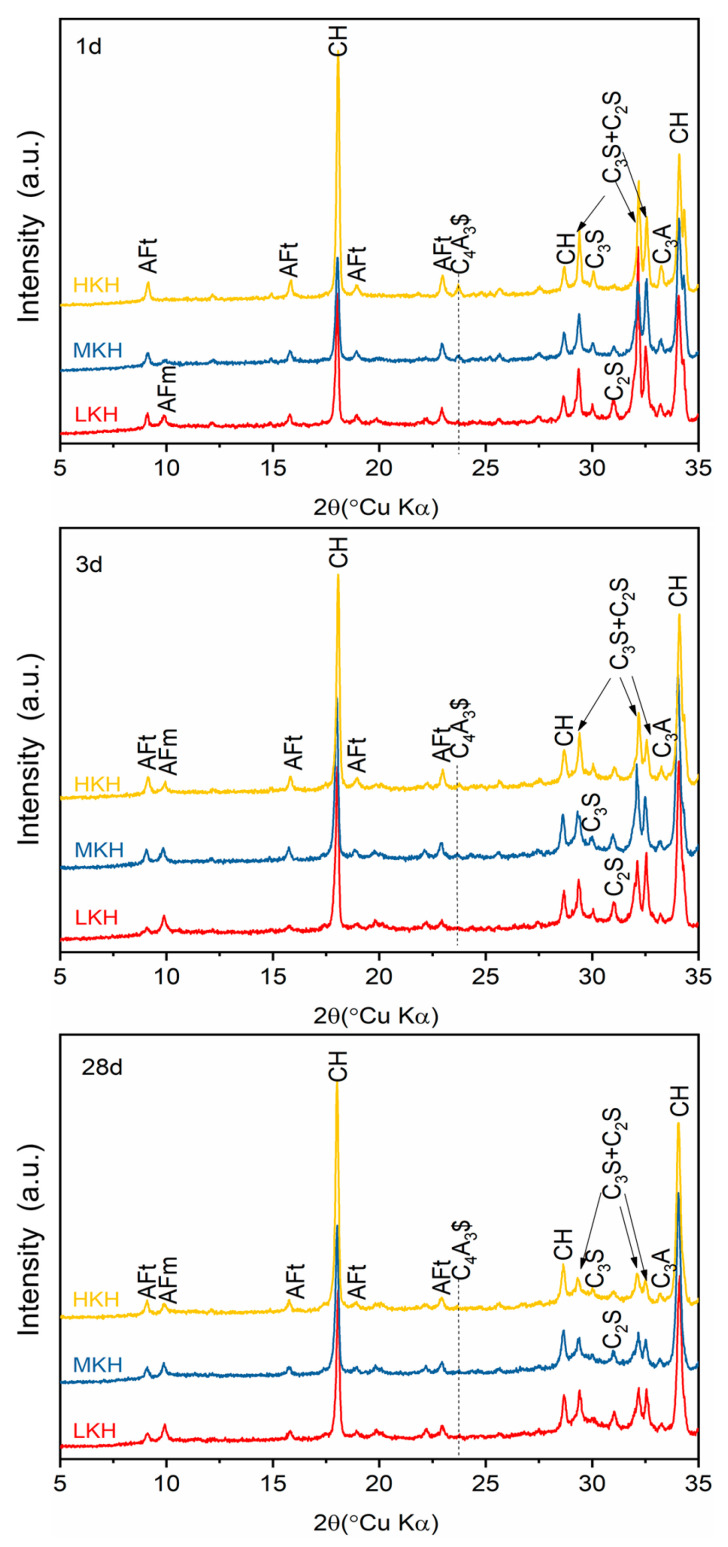
XRD spectra of hydration pastes at 1, 3, and 28 d.

The hydration products of AC$A cements primarily consist of Portlandite, AFt, and AFm. Portlandite and AFt can be detected at the early stages of hydration, while AFm typically appears 3 days after gypsum depletion (Figure 6). AFm-type phases are the main hydrated crystalline phases of ye’elimite [34]. In LKH, AFm is detected after 1 day, coinciding with the complete consumption of C_4_A_3_$, whereas in MKH and HKH, C_4_A_3_$ mostly hydrates within 3 days and disappears by 28 days [25]. The decrease in the AFt peak observed in the LKH-3d spectrum supports the formation of AFm through the transformation of AFt [24,35]. After 28 days of hydration, nearly all C_3_S has reacted, while C_2_S remains unreacted (as evidenced by the peaks near 30° in Figure 6).

**Figure 7:**
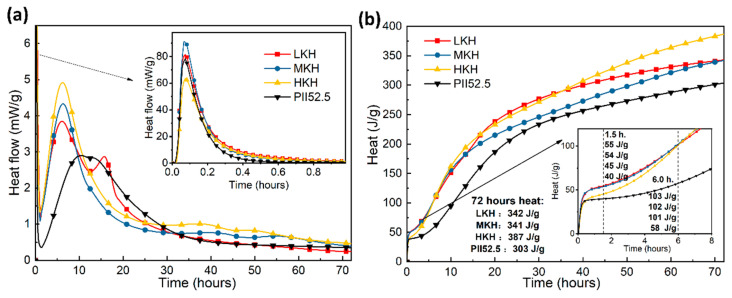
Heat flow (**a**) and total heat release (**b**) of ACSA clinkers.

Figure 7 illustrates the heat evolution of AC$A and PII 52.5 cements. On the heat flow plot, the initial exothermic processes of cement dissolution and early-stage reactions, particularly the formation of AFt, are represented by the first peak observed within half an hour. The primarily exothermic peak is attributed to the rapid formation of C-S-H and CH resulting from the hydration of C_3_S. The shoulder peak, or third peak, corresponds to the formation of AFm from AFt (Figure 7a) [24]. Among the samples, HKH, which has the highest C_3_S content, exhibits the lowest dissolution peak and the highest peak for C-S-H formation. As the KH value increases, the peak for AFm formation gradually transitions from a third peak around 15 h hydration for LKH to a small shoulder peak at 10 h and bulge after 30 h of hydration for MKH, and eventually only bulges after 30 h of hydration for HKH. This observation is consistent with the XRD data presented in Figure 6. In general, AC$A cements display faster hydration within 72 h compared to PII 52.5 cement due to the presence of ye’elimite [25]. The total heat release of AC$A cement (341–387 J g^−1^) is 12.5–27.7% higher than that of PII 52.5 cement (303 J g^−1^) (Figure 7b).

**Figure 8:**
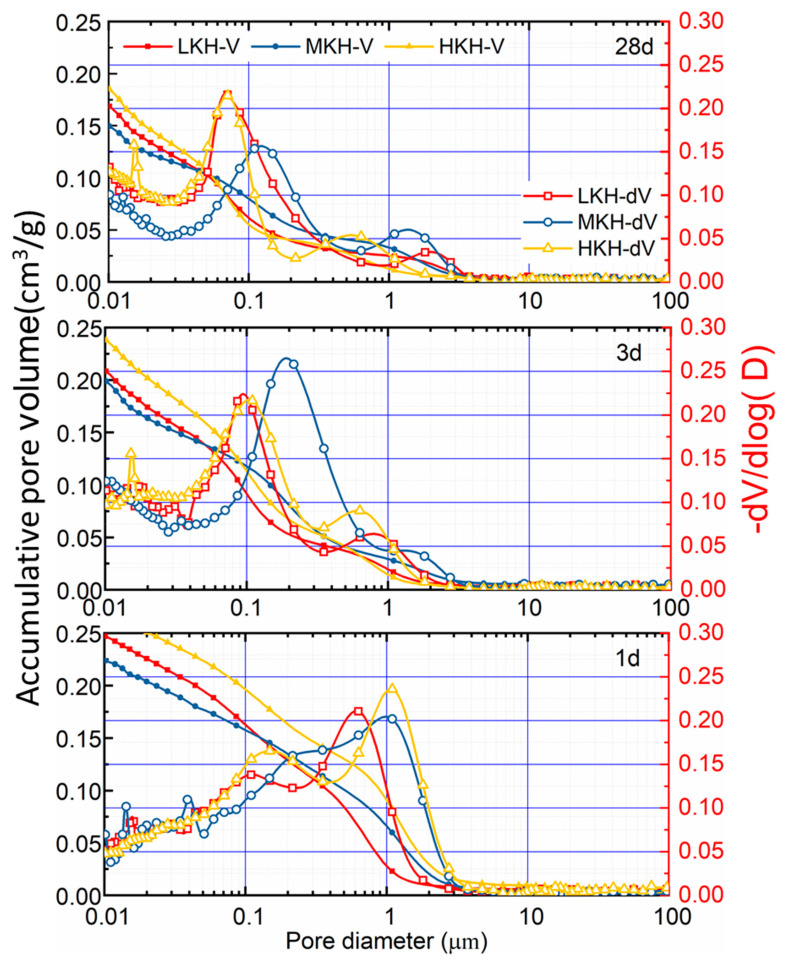
Pore volume and porosity distribution of pastes at 1, 3, and 28 d.

The pore structure of the hydrated pastes was probed by mercury intrusion porosimetry. The porosity changes with time (1, 3, and 28 days) were due to the loss of free water and the formation of hydration products. As shown in Figure 8, the pastes possessed a pore size distribution from 0.01 to 10 μm, with two peaks on each differential distribution curve corresponding to micron and sub-micron pores. The accumulative pore volume decreased from 0.22~0.3 to 0.15~0.18 cm^3^ g^−1^, as the hydration time increased. Meanwhile, the pore size is finer and more narrowly distributed via hydration than the main peak of the pore diameter that is left-shifted with the value decreasing about one order of magnitude.

The cumulative pore volume of the MKH paste consistently remains lower than the other pastes throughout the hydration process. This confirms the higher compressive strength of MKH at 1, 3, and 28 days. On the other hand, the overall aperture distribution of LKH after 28 days of hydration is concentrated at smaller pore diameters, contributing to its higher compressive strength at that time point (Figure 5). In contrast, HKH exhibits the opposite trend, with a higher pore volume and lower compressive strength. It is worth noting that both the porosity and pore aperture distribution of the pastes significantly influence the mechanical properties of cementitious materials. The 28-day-hydrated LKH paste demonstrates a more concentrated distribution of smaller pore diameters, resulting in a higher compressive strength compared to the other pastes at 28 days, as depicted in Figure 5.

**Figure 9:**
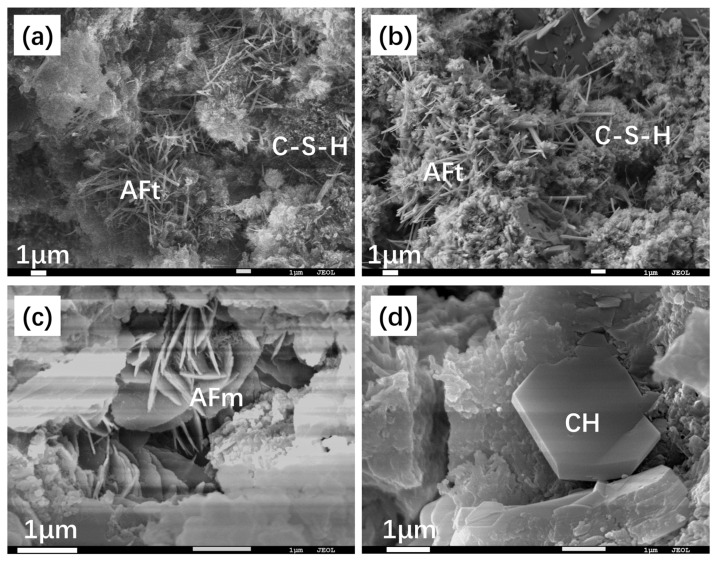
SEM images of MKH hydration pastes at 1 d (**a**), 3 d (**b**), and 28 d (**c**,**d**).

The SEM images in Figure 9 illustrate the hydration process of MKH at 1, 3, and 28 days. At early stages of hydration (Figure 9a,b), needle-shaped AFt crystals are observed, which later combine with the C-S-H gel to form a composite crystal–gel network structure. This structure significantly contributes to the high early strength of the AC$A cement, as evidenced in Figure 5. In the 28-day hydrated paste, petaloid-shaped thin plates of AFm are visible in the voids (Figure 9c). These thin plates represent a transformed AFt phase into AFm, which occurs after the sulfur in the matrix has been depleted. This transformation is consistent with the XRD results depicted in Figure 6. Additionally, Figure 9d shows the presence of hexagonal plate-shaped CH and thin plate-shaped AFm, which help in dividing large pores into smaller ones with a sub-micron scale size [25]. This observation aligns with the results obtained from MIP analysis shown in Figure 8. Moreover, Figure 9d demonstrates that the paste densifies after 28 days of hydration, contributing to the development of late strength as observed in Figure 5.

## 4. Conclusions

The following conclusions can be drawn from this work on the impact of lime saturation factor (KH) on the synthesis and hydration of AC$A:

(1) A great amount of C_3_S (64.88%) and C_4_A_3_$ (2.06%) can coexist in the resulting clinker with the sintering temperature at 1470 °C and secondary heat treatment temperature at 1270 °C, while the content of f-CaO is qualified.
(2) Increasing KH leads to higher C_3_S content and lower C_2_S content, while the presence of ye’elimite shows minimal correlation with KH. Heat release shows that as the KH value increases, the peak for AFm formation gradually obscures. In this study, based on the phase components in the clinker and mechanical strength of the mortar, the MKH labeled sample with a KH value of 0.93 was chosen for optimizing AC$A clinker sintering.
(3) AC$A paste showed significantly improved compressive strength development during the early curing period than PII 52.5 due to the rapid hydration of C_4_A_3_$ to form needle-shaped AFt crystals. These crystals then combine with the C-S-H gel to create a crystal–gel network structure. MKH exhibits higher compressive strength at 1, 3, and 28 days, which can be confirmed by the pore structure analysis revealing consistently lower accumulative pore volume in MKH paste compared to other pastes.

## Figures and Tables

**Table 1:** Composition of each raw material (wt.%).

Composition	LOSS	SiO_2_	Al_2_O_3_	Fe_2_O_3_	CaO	MgO
Limestone	43.31	0.34	0.24	0.25	54.93	0.13
Fly ash	1.96	54.15	31.49	3.38	1.98	1.27
Clay	17.02	30.75	31.24	15.32	2.35	0.65
Silica	0.36	93.95	2.38	1.96	0.48	0.32

**Table 2:** The chemical components of raw meals (wt.%).

Label	KH	LOSS	CaO	SiO_2_	Al_2_O_3_	Fe_2_O_3_	MgO	MgO *	K_2_O *	SO_3_ *
LKH	0.90	35.07	43.48	14.25	4.32	1.39	0.26	2.00	0.80	3.00
MKH	0.93	35.25	43.73	13.95	4.22	1.36	0.26	2.00	0.80	3.00
HKH	0.96	35.42	43.98	13.65	4.13	1.33	0.25	2.00	0.80	3.00

* The weights of MgO, K_2_O, and SO_3_ were calculated as exogenous ingredients in the raw meal.

**Table 3:** Mineral compositions of the clinkers calculated by XRD Rietveld quantification (%).

Label	C_3_S	C_2_S	C_3_A	C_4_AF	f-CaO	f-MgO	C_4_A_3_$	Anhydrite	R_wp_
LKH-1470	61.86	22.99	10.14	1.73	0.40	2.21	0.32	0.33	10.82
LKH-1470-1270	53.42	26.18	8.25	5.90	0.29	3.61	2.06	0.28	11.8
MKH-1470	67.27	14.92	9.95	3.33	0.31	2.77	1.09	0.37	10.99
MKH1470-1270	64.88	14.46	9.88	4.89	0.45	3.24	2.06	0.15	9.58
HKH-1470	75.35	7.2	9.59	2.69	0.92	2.47	1.44	0.34	11.12
HKH-1470-1270	68.22	9.41	9.29	5.46	0.53	3.64	2.86	0.48	10.41

## Data Availability

The original contributions presented in the study are included in the article, further inquiries can be directed to the corresponding authors.
